# Association of susceptible genotypes to periodontal disease with the clinical outcome and tooth survival after non-surgical periodontal therapy: A systematic review and meta-analysis

**DOI:** 10.4317/medoral.20638

**Published:** 2015-11-22

**Authors:** Georgios-Sokratis Chatzopoulos, Aikaterini-Ellisavet Doufexi, Fotini Kalogirou

**Affiliations:** 1 DDS, Resident, Advanced education program in Periodontology, University of Minnesota, Minneapolis, Minnesota, USA; 2 DDS, PhD, Private practice limited to periodontics and implant dentistry; 3 PhD, MSc, BSc, RN, Nursing Department, School of Health Sciences, Cyprus University of Technology, Limassol, Cyprus

## Abstract

**Background:**

The real clinical utility of genetic testing is the prognostic value of genetic factors in the clinical outcome of periodontal treatment and the tooth survival. A meta-analysis was undertaken to estimate the effect of a susceptible genotype to periodontitis on the clinical outcomes of non-surgical periodontal therapy and the tooth survival.

**Material and Methods:**

A systematic search of MEDLINE-Pubmed, Cochrane Library and Scopus was performed. Additionally, a hand search was done in three journals. No specific language restriction was applied. Two reviewers screened independently titles and abstracts or full text copies. Quality assessment of all the included studies was held.

**Results:**

Initial screening of electronic databases resulted in 283 articles. Ten studies met the inclusion criteria, nine of them examined the clinical outcome, while the other one investigated the tooth survival in susceptible individuals after non-surgical periodontal therapy. Eight of included studies were selected for the meta-analysis. IL-1 positive genotypes increase the risk of tooth loss, while no association found between the bleeding on probing (BOP), clinical attachment loss (CAL) and plaque index (PI) with the genotype status. Probing pocket depth (PPD) reduction in the first three months and in long-term results found to have a significant association with the genotype.

**Conclusions:**

There is no difference in the clinical measurements after non-surgical periodontal treatment, apart from PPD. More publications are needed to identify a cause-effect relationship.

**Key words:**Periodontal disease, periodontitis, periodontal therapy, clinical outcome, tooth loss, susceptibility, polymorphism, genotype, meta-analysis, systematic review.

## Introduction

Periodontal disease is commonly defined as a chronic multifactorial infectious disease where the tissue supporting the teeth is destroyed (1). It was believed that the progression of periodontitis is a result of microbial and environmental factors. Nowadays there is evidence supporting that genetic susceptibility plays a role in the onset and progression of periodontitis (2,3). The existence of high-risk group of patients could not be explained by the microbiology alone (4). Approximately 10-15% of the population appears to have quickly progression from gingivitis to periodontitis (4). As in other complex diseases is estimated that 10 to 20 genes are involved in the onset and progression of periodontal disease. Ethnic populations appear to have different alleles encoding the same gene (5).

The researchers are seeking genetic evidence to explain the differences in periodontal disease susceptibility. The first evidence that genetics play a role in periodontal disease appeared in the early 1990s. The presence of a genetic risk factor increases the probability of developing periodontal disease (6-8). Cytokines as Interleukin-1 (IL-1), Interleukin-2 (IL-2), Interleukin-4 (IL-4), Interleukin-6 (IL-6), Interleukin-10 (IL-10), Tumor necrosis factor (TNF), Transforming growth factor-β1 (TGF-β1), cell-surface receptors, chemokines and enzymes are proteins translated from different DNA sequences (9). All of them play determinant roles in antigen recognition, immune system and host response (9).

Gene polymorphisms

IL-1 is a pro-inflammatory cytokine which plays an important role in chronic inflammation and has been implicated in chronic diseases, such as periodontitis (10). A combined genotype with single nucleotide exchanges in the IL-1A and IL-1B gene was found to be associated with an increased risk of periodontitis (11).

IL-4 is another inflammatory cytokine which is a potent down regulator of macrophage function. Lack of IL-4 in periodontal tissues may cause increased CD14 expression and high production of IL-1B, TNF-a and PGE2 in human monocytes with an end result of bone resorption (12). Polymorphism in the IL-4 gene has been investigated and it has been reported that individuals-carriers of the TCI/CCI haplotype are more susceptible to chronic periodontitis than those who carry the TTD/CTI haplotype. The haplotype T(-590)/T(-33)/allele 2 VNTR (70 base pairs) (2) of the IL-4 gene was significantly more frequent in patients with chronic periodontitis (13).

Interleukin-8 is a cytokine which activates the neutrophils (14). Studies have found that the SNPs in the IL-8 gene like -251 (T/A), +396 (T/G), +781 (C/T) were associated significantly with the presence and severity of chronic periodontitis (15,16).

Matrix metalloproteinases (MMPs) are host and bacterial derived proteinases (17). MMPs play an important role in wound healing (18). MMPs degrade different proteins of the extracellular matrix, for example different types of collagens. As a result matrix metalloproteinases can determine the inflammatory response (17). MMP-1 and MMP-13 gene polymorphisms have found to influence the levels and the activity of MMPs which in turn are associated with the progression of periodontal disease (19). Several polymorphisms have been well characterized and recognized an association or not with chronic periodontitis (20).

Mannose binding lectin (MBL) is a protein with important role in innate immunity. MBL is associated with the first line of defense against infection. Many studies have found an influence of MBL on various bacterial diseases including periodontal disease (21). It has been found two types of MBL genes: MBL-1 which is a pseudogene and MBL-2 (22).

The main purpose of periodontal therapy is the long term retention of natural teeth in a healthy environment. Identifying gene polymorphisms can contribute in diagnostics for susceptibility to periodontal disease (9). Nowadays, the real clinical utility of genetic testing is the prognostic value of genetic factors in the clinical outcome of periodontal treatment and the tooth survival.

The purpose of this paper was to assess the clinical outcomes of non-surgical periodontal therapy and the tooth survival after non-surgical therapy in patients with genetic susceptibility to periodontal disease.

## Material and Methods

The systematic review was conducted according to the Cochrane handbook for systematic review (23). The transparent reporting of systematic reviews and meta-analyses has been ensured by the use of the PRISMA- Statement (24).

* Review questions

- The following questions were addressed:

What is the effect of a susceptible genotype of periodontitis following non-surgical periodontal therapy on the clinical parameters of periodontal inflammation, such as probing pocket depth (PPD) measurements, clinical attachment level (CAL) measurements, bleeding on probing (BOP) score, plaque index (PI), gingival bleeding index (GBI) and on the survival of teeth, compared to non-susceptible genotypes?

What is the influence of smoking habits on the clinical outcomes after non-surgical periodontal therapy in individuals who are susceptible to periodontitis?

- Literature search

Eleven key terms were used, combined in three search strategies. The electronic databases were electronically searched using the following search strategy:

1) (periodontal therapy OR clinical outcome OR tooth loss)

2) (periodontal diseases OR periodontitis OR periodontal disease)

3) (polymorphism, genetic OR polymorphism OR genotype OR haplotype)

4) 1 AND 2 AND 3.

These sources included the US National Library of Medicine National Institutes of Health (MEDLINE-Pubmed), the Cochrane Library and Scopus (Database by Elsevier). Articles from inception of these databases up to the end of March 2014 (week 4) were considered. A second search in the same sources was conducted before the statistical analysis was completed in order to make sure that no new articles were published. A complementary manual search up to the same period was carried out in the following Journals: Journal of Clinical Periodontology, Journal of Periodontology and Journal of Periodontal Research. No specific language restriction was applied to any of the searches, although the electronic searches could only identify studies including at least a title in English because the terms were in English.

- Study selection

Abstracts were obtained for all the titles identified during electronic and manual searches. If eligibility aspects were present in the title, then the study was referred for further reading. If none of the aspects were present, then the abstract was read in details. Full-text copies were obtained for all titles remaining after screening. Two reviewers screened independently titles and abstracts or full text copies to eliminate articles that clearly failed to meet the inclusion criteria:

1) Papers written in any language.

2) Studies conducted in humans, aged 18 years and older, in good general health and diagnosed with chronic periodontitis.

3) Studies investigate the influence of susceptible genotypes of periodontitis on the clinical outcome or on the number of tooth loss after non-surgical periodontal treatment.

4) Evaluation of the clinical outcome with one or more of the clinical parameters: probing pocket depth (PPD), clinical attachment level (CAL), bleeding on probing scores (BOP), plaque index (PI), gingival bleeding index (GBI).

5) Evaluation of the tooth loss with the number of teeth before and after non-surgical periodontal treatment .

6) Baseline measurements and final measurements of clinical parameters at least four (4) weeks after non-surgical periodontal treatment is needed.

7) Type of therapy: Non-surgical periodontal treatment of scaling and root-planing performed by periodontist/periodontists.

Exclusion criteria included surgical periodontal treatment and/ or treatment with the use of antibiotics.

In case of disagreement, it was resolved by discussion among the two reviewers. The articles that fulfilled the above criteria were processed for data extraction.

- Data extraction

The data from articles that met the inclusion criteria were extracted by the two reviewers. The clinical parameters that were used to evaluate the periodontal treatment outcome were at least one of the PPD, CAL, BOP, PI, GBI. The tooth survival was measured by the number of teeth pre-treatment and post-treatment. For studies that presented more than one outcome assessment, only the baseline and the final measurements were used.

- Quality assessment

The quality assessment of all the included studies in this meta-analysis was performed based on the Newcastle–Ottawa scale (NOS) (25). The modified NOS was used as described by Chambrone *et al*. (26) and assessment of selection, comparability, exposure and statistical bias were performed. In our study additional changes were performed to Chambrone’s modified NOS in order to assess the methodological quality of the included studies. All the methodological quality assessment criteria that were utilized in this study are presented in  Table 1.

**Table 1:**
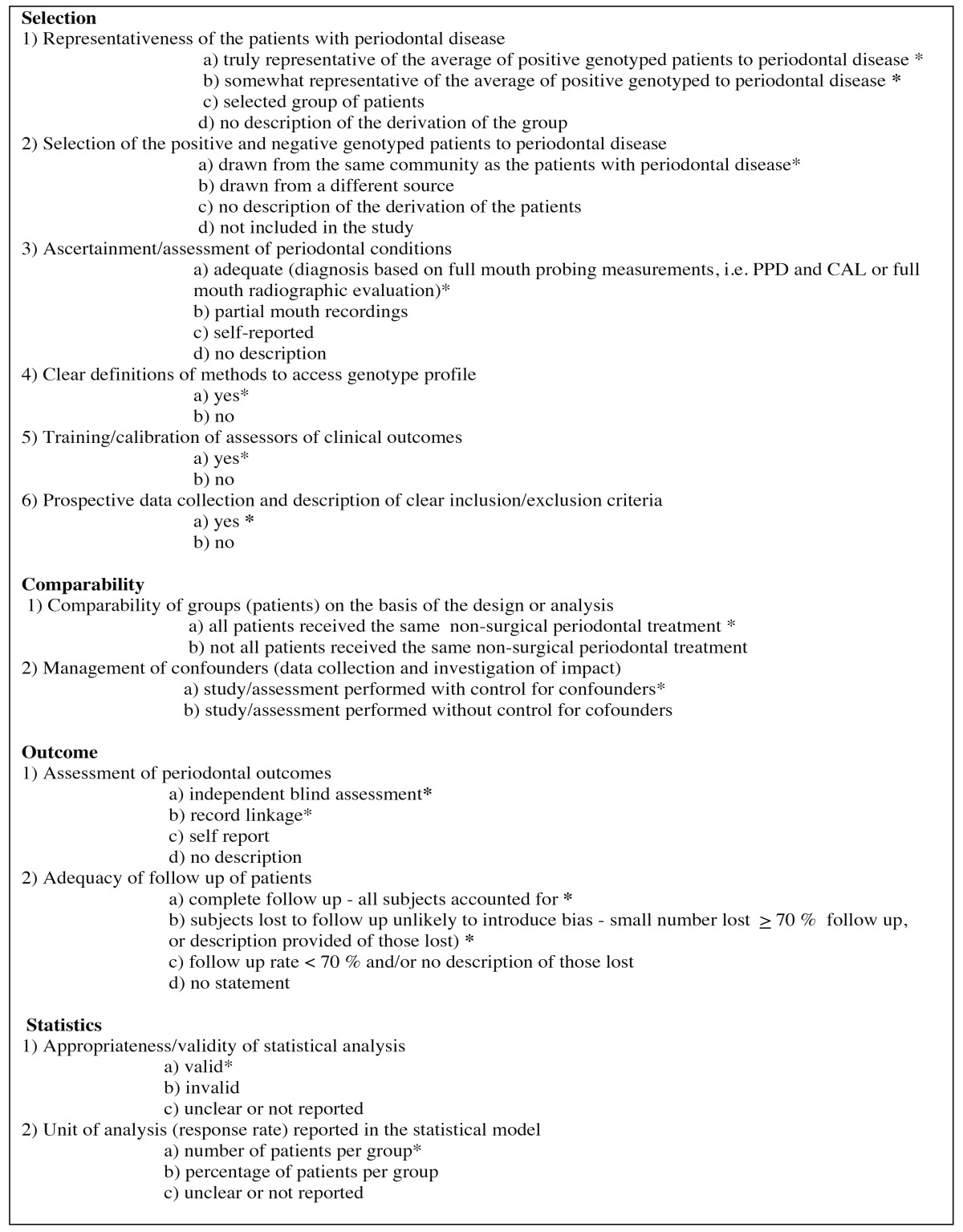
Quality assessment using the modified Newcastle-Ottawa Scale (NOS).

- Statistical analysis 

Analyses were performed according to the availability of data for each of the outcomes (PPD, CAL, BOP, PI), for several post-enrollment time frames. The measure effect was thus expressed and presented as standardised mean difference of the improvement (change) observed within three different time intervals: one month / 45 days, three months and six months. The main analysis, which included all of the studies, was based on their long-term results, whereas individual studies were grouped according to their maximum follow up period (subgroup analysis). Before proceeding to the analyses, further calculations were deemed necessary. These included the transformation of values expressed in the form median (minimum – maximum) into the form of mean (standard deviation - SD) (27). Changes in means were calculated by subtracting baseline from follow up values, within each group of individual studies. SDs of the changes in means, were then calculated by using a formula, based on the assumption that the correlation coefficient [r] between the two measures is equal to 0.5. Pooled estimates and 95% confidence intervals were calculated by using an inverse variance random-effects approach, for each of the outcomes. Reanalysing data using a fixed-effects model, yielded similar results. Results are presented in the form of forest plots. Heterogeneity was explored by using the I2 statistic and chi-square test. The small number of included studies did not allow further assessment of heterogeneity for identifying its possible sources. Funnel plots were used to detect potential publication bias in regard to the long term effects of each study. All analyses were performed using the Rev Man 5.3 software.

## Results

**Figure 1:**
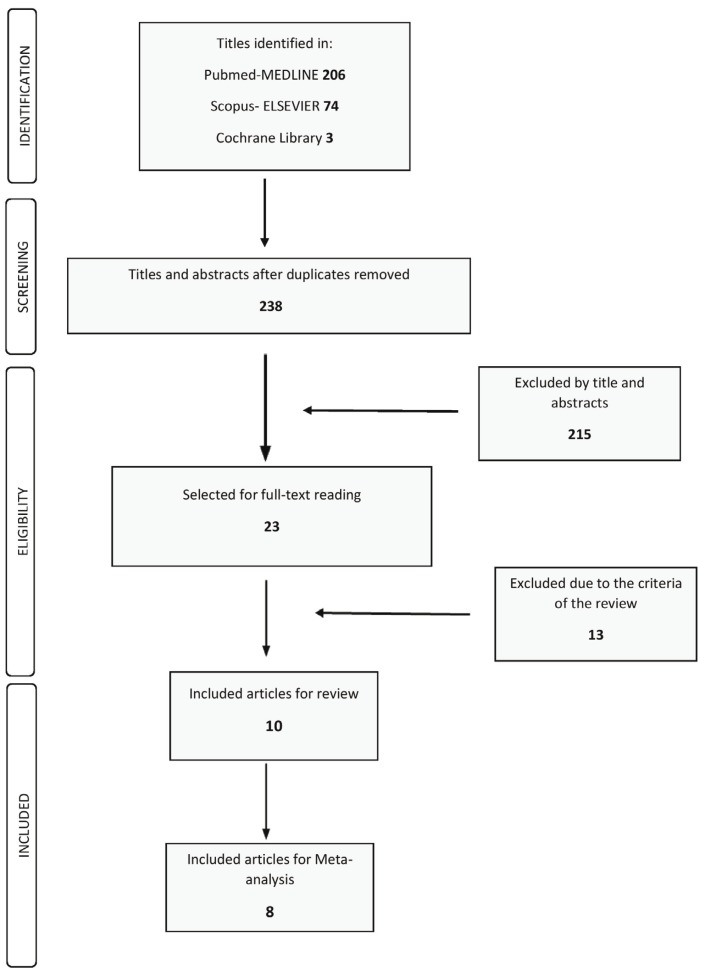
Study selection.

Initial screening of electronic databases resulted 283 articles; 206 of them were retrieved from the search of Pubmed-MEDLINE, 74 from Scopus-Elsevier and 3 from Cochrane Library. The screening of titles and abstracts initially resulted 23 articles. Based on inclusion and exclusion criteria 13 of them were excluded (Fig. 1). The 13 studies were excluded for the following reasons: Surgical treatment in some cases and maintenance (28-35); surgical treatment in some cases and patients accepted to be <18 years old (36,37); anti microbial therapy with antibiotics (38); periodontal therapy performed by hygienist (39); not systematically healthy patients (39); sample consisted of patients with aggressive periodontitis (40). Hand searching of the selected journals did not reveal any additional publication.

The final selection included 10 articles (Fig. 1). In 9 of these studies the influence of susceptible genotypes of periodontitis on the clinical outcome was investigated (22,41-48) while one of them examined the number of teeth lost after non-surgical periodontal treatment in susceptible and non-susceptible patients to chronic periodontitis (49). We contacted the authors of two studies to obtain details for the study sample size and the PPD baseline measurements that were performed by the examiners. One author did not reply and the study was excluded. The other author provided the important details and the article included in our study.

- Study Characteristics

The characteristics of the included studies in the present systematic review are presented in  Table 2, Table 3.

**Table 2:**
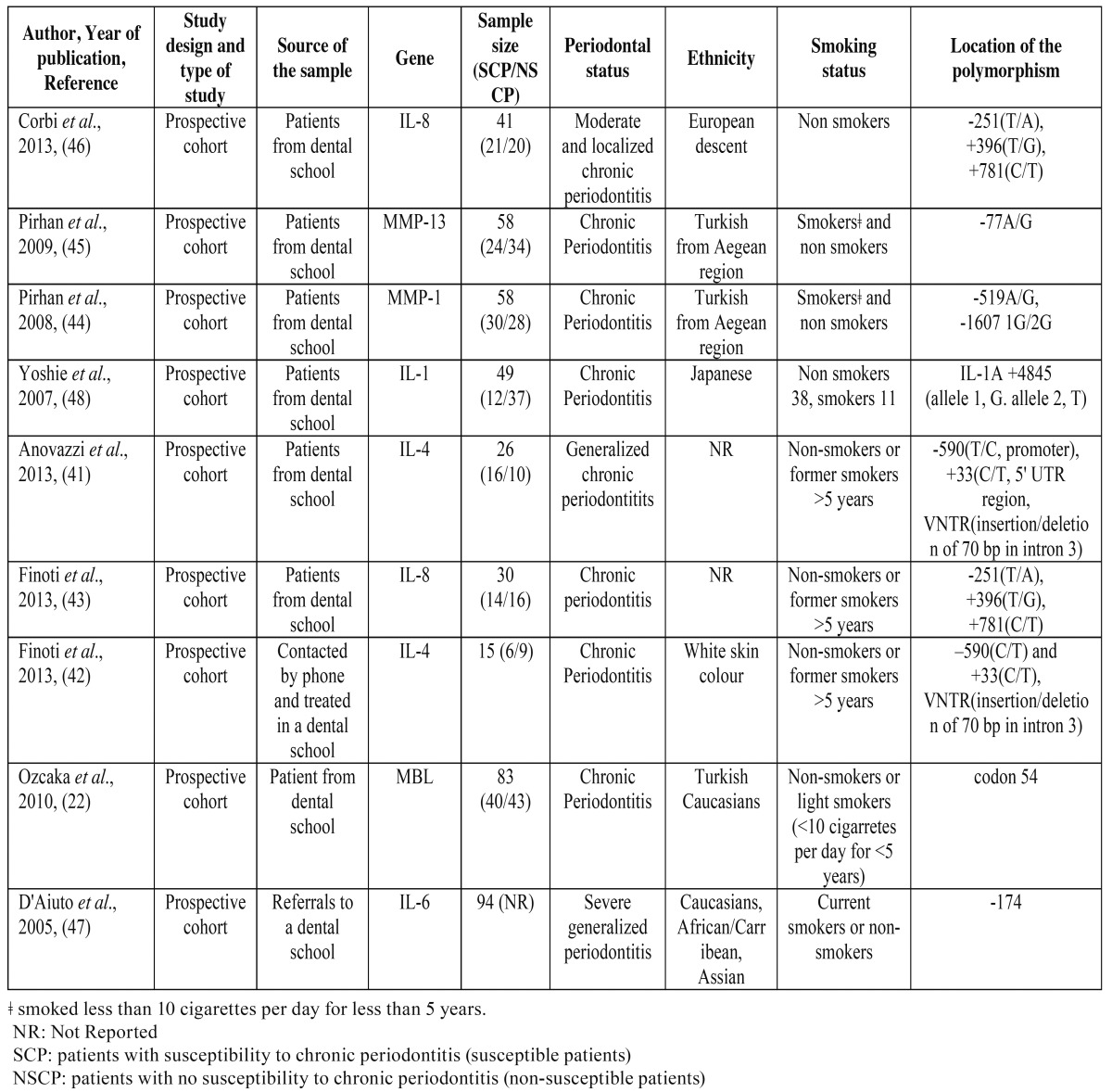
Main characteristics of the included studies of the systematic review investigating the association between the genotype status and the clinical outcomes of the non-surgical periodontal therapy.

**Table 3:**
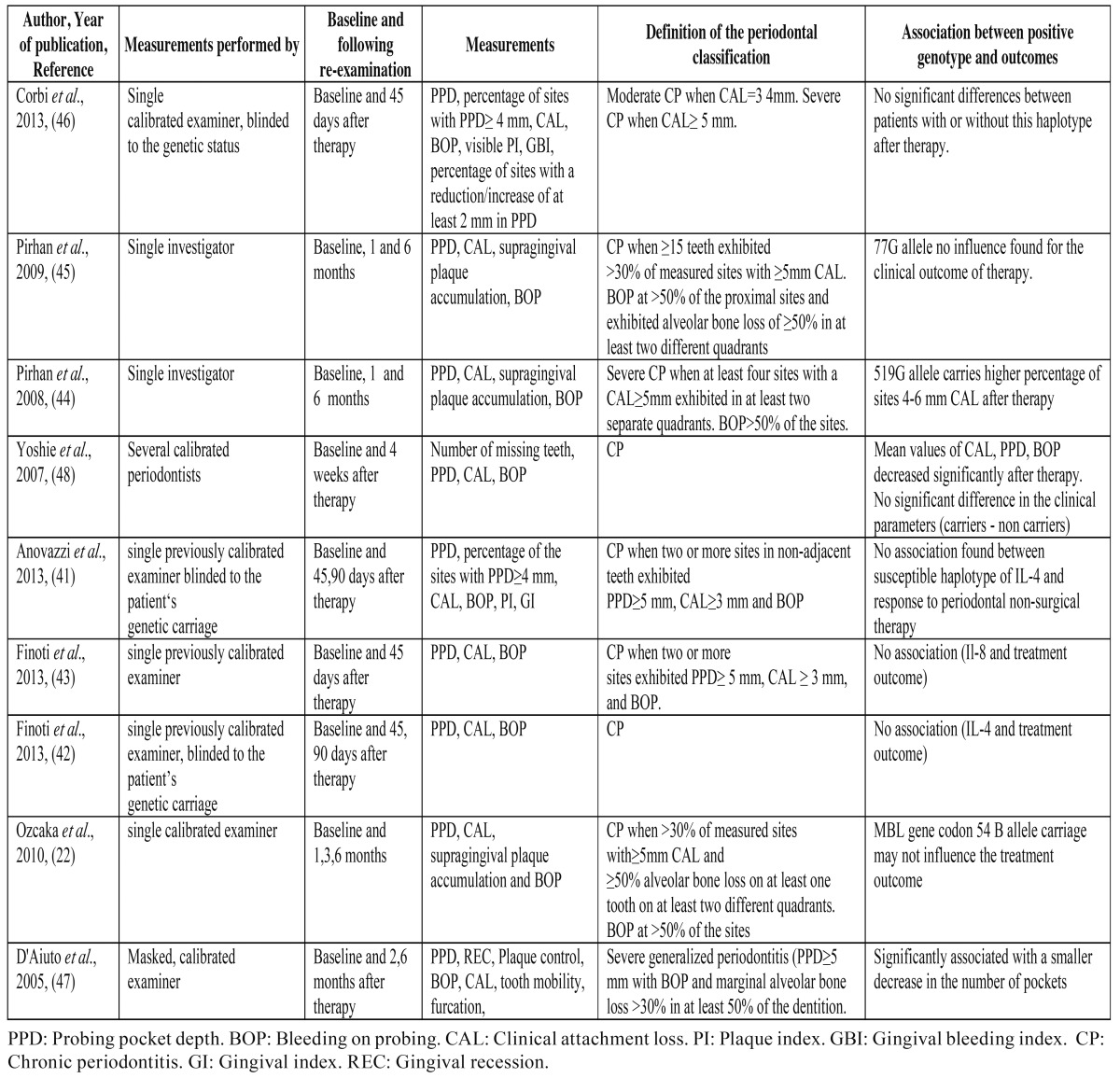
Characteristics of the study design of the included studies of the systematic review investigating the association between the genotype status and the clinical outcomes of the non-surgical periodontal therapy.

The year of publication of the selected studies range from 2005 to 2013. The sample included patients diagnosed with chronic periodontitis. Also, patients with healthy periodontal tissues were included in five studies in order to meet other objectives (22,41,42,44,45). In case of chronic periodontitis, non-surgical periodontal treatment with oral hygiene instruction, scaling and root planing under local anesthesia, was performed by one or more periodontists. The sample was selected to be systematically healthy. Patients’ ethnicity varied among: European descent, Turkish from Aegean region, Japanese, White skin colour, Turkish Caucasians, Caucasians, Africans/Carribeans and Asians. Measurements were performed at least one month after baseline examination in all the included studies.

A wide range of gene polymorphisms was studied and Interleukins’ polymorphisms are the most investigated. In the included studies of this meta-analysis, the association between IL-1, IL-4, IL-6, IL-8, MMP-1, MMP-13 and MBL haplotypes and the clinical outcomes was investigated. Probing pocket depth (PPD), Clinical attachment level (CAL) and Bleeding on probing (BOP) were at least measured in the nine articles that studied the clinical outcome of non-surgical periodontal therapy. McGuire and Nunn (49) measured PPD, presence of furcation, mobility, bone loss and the crown to root ratio. The number of subjects in each study ranged from 15 (42) to 94 patients (47).

The majority of the included studies consisted of nonsmokers or more than five years former smokers. The evaluation of the influence of smoking habits on the clinical outcomes of susceptible to chronic periodontitis patients was one of the goals of this meta-analysis. The sample size of smokers was extremely, low while the lack of data of smokers and the absence of statistical analysis between the smoking habits and the periodontal therapy outcomes made impossible to conclude to a measurable result. Only one study examined this parameter. Smokers (9 smokers, 30 history of smoking and 3 nonsmokers) were included in the retrospective study of McGuire and Nunn (49) and after 14 years of the baseline measurements, they concluded that IL-1, smoking and the combined effect increase the risk of tooth loss. The sample of this study was patients from a private practice with a mean age of 46 years and the age ranged from 33 to 62 years. IL-1 positive subjects was the 38.1% of the total sample.

Analyses were stratified according to the clinical parameters (BOP, PPD, CAL, PI) of the susceptible and non-susceptible patients to periodontal disease and the time of the follow-up measurements. Results from the random effects model are reported. To assess heterogeneity, a *p*-value, derived from Cochran’s Q statistics for the null hypothesis was calculated.

All the included studies in this systematic review were assessed by the use of the modified NOS regarding the study design (selection, comparability, outcome and statistics). Bias assessment revealed that all of the included studies were of high quality (range from 10 to 12 points). The scores of the methodological quality assessment of the studies selected for this review are presented in  Table 4.

**Table 4:**
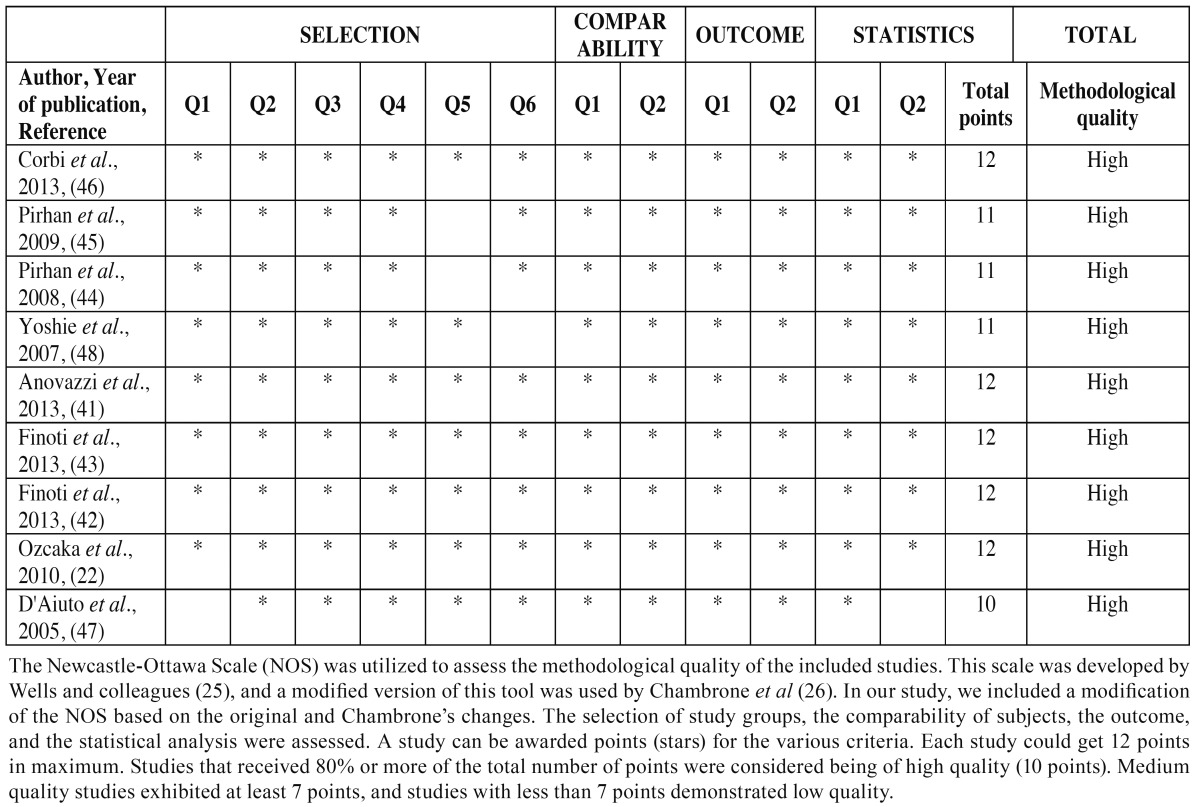
Quality assessment of the included studies.

- Baseline up to 30 or 45 days after initial examination

Firstly the analysis was conducted for the studies with a follow-up of 30 to 45 days and evaluated the differences between the baseline and the 30 or 45 days measurements. The random effects estimate for the mean difference of the BOP scores was -0.03% [95% CI: -0.31, 0.26], the mean difference of the CAL was 0.04mm [95% CI: -0.17, 0.25], the mean difference of the PI scores was -0.05% [95% CI: -0.29, 0.19], while the mean difference of the PPD was -0.09mm [95% CI: -0.29, 0.11]. There is no evidence for a significant difference in the evaluated parameters when comparing susceptible and non-susceptible patients.

- Baseline up to 3 months after initial evaluation

The random effects estimates for the mean differences between the initial and the 3 months measurements of the BOP scores, CAL, PI scores and PPD were 0.12% [95% CI: -0.26, 0.50], -0.29mm [95% CI: -0.78, 0.21], 0.24% [95% CI: -1.03, 1.50] and -0.65mm [95% CI: -1.14, 0.16] respectively. Measurements of BOP scores and PI scores were included in only in two studies (22,41) while measurements of CAL and PPD were presented in three studies (22,41,42) and the meta- analysis could not provide a safe result.

- Baseline up to 6 months after initial evaluation

Four groups of patients from three different studies (22,44,45) were reexamined six months after the initial examination. The meta-analysis did not provide evidence for statistical significance between the SCP and NSCP groups in regard to the change from baseline to final measurements. The random effects estimates for the mean differences between the initial and the 3 months measurements of the BOP scores, CAL, PI scores and PPD were -2.64% [95% CI: -11.62, 6.34], -0.09mm [95% CI: -0.34, 0.15], -0.14% [95% CI: -0.39, 0.11] and -0.18mm [95% CI: -0.43, 0.06] respectively.

- Long term results of the studies with subgroup analysis according to time periods (30-45 days, 3 months and 9 months)

Results of the meta-analysis did not reveal any systematic significant differences in measure effect that would favor a specific genotype profile against another, with regard to their response to treatment, for each of the BOP, CAL and PI scores. The *p*-values were calculated as 0.33, 0.74 and 0.96 respectively. The mean differences were estimated using random effects models: -0.14% [95% CI: -0.44, 0.15] for BOP, -0.03mm [95% CI: -0.24, 0.17] for CAL and -0.01% [95% CI: -0.35, 0.34] for PI scores. As far as PPD measurements are concerned, the *p*-value was found to be statistically significant, (*p*= 0.03), with I2=0% which indicates absence of heterogeneity and mean differences of -0.22mm [95% CI: -0.41, -0.02].

**Figure 2:**
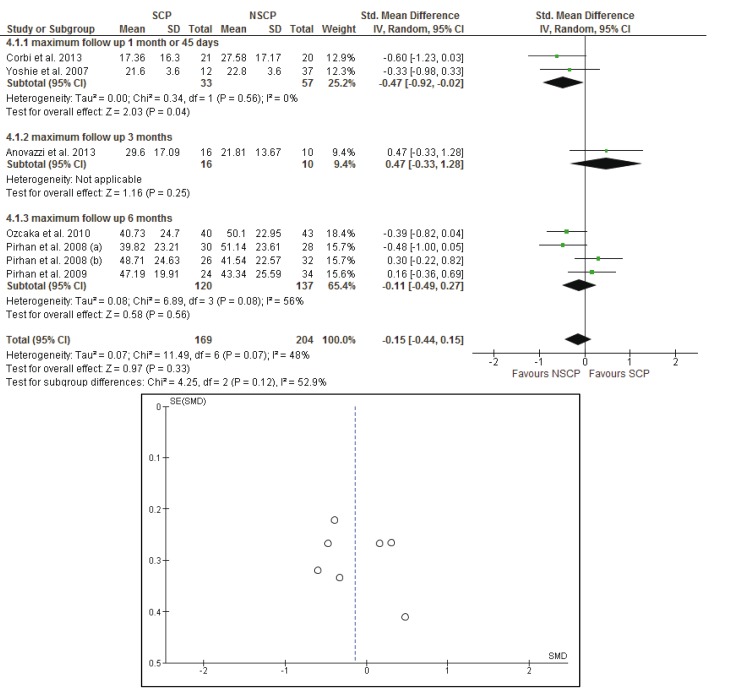
The forest plot and the funnel plot of the comparison: long term effects of each study and BOP (%). Meta-analysis and forest plot comparing susceptible (SCP) and non-susceptible (NSCP) to periodontal disease with the difference of bleeding on probing score (BOP) long term. Presenting the mean differences of bleeding on probing (BOP) after non-surgical periodontal therapy in 1 month-45 days, 3 months, 6 months separately and as total. BOP is measured in percentage. The funnel plot is designed to check for the existence of publication bias.

**Figure 3:**
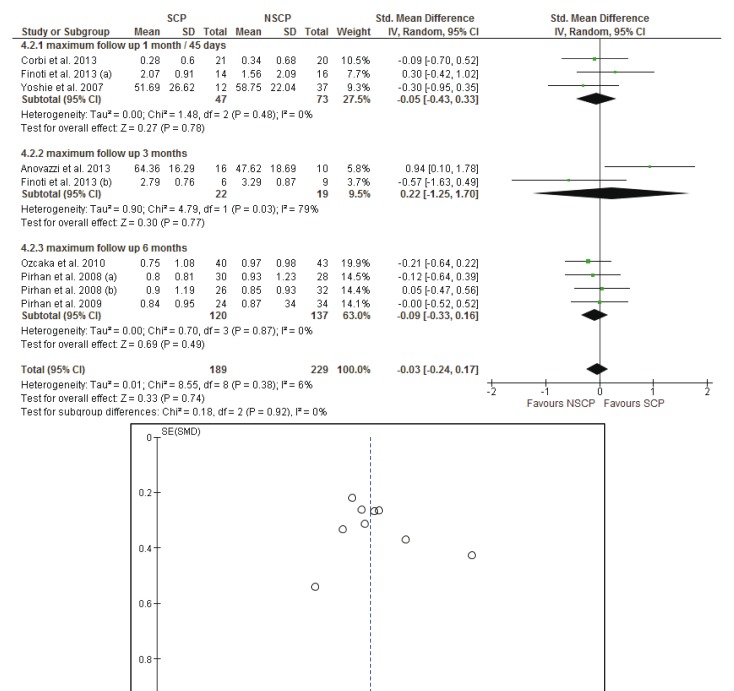
The forest plot and the funnel plot of the comparison: long term effects of each study and CAL (mm). Meta-analysis and forest plot comparing susceptible (SCP) and non-susceptible (NSCP) to periodontal disease with the difference of clinical attachment loss (CAL) measurements long term. Presenting the mean differences of clinical attachment loss (CAL) in 1 month-45 days, 3 months, 6 months separately and as total after non-surgical periodontal therapy. CAL is measured in millimeters (mm).A funnel plot is a graph designed to check for the existence of publication bias.

**Figure 4:**
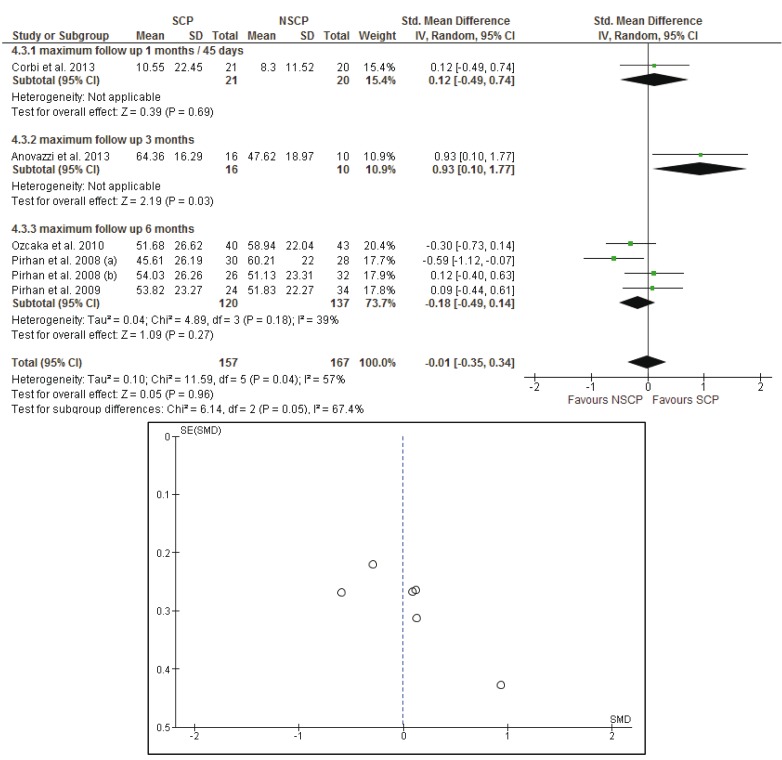
The forest plot and the funnel plot of the comparison: long term effects of each study and PI (%). Meta-analysis and forest plot comparing susceptible (SCP) and non-susceptible (NSCP) to periodontal disease with the difference of plaque score (PI) long term. Presenting the mean differences of plaque index (PI) after non-surgical periodontal therapy in 1 month-45 days, 3 months, 6 months separately and as total. PI is measured in percentage. The funnel plot is designed to check for the existence of publication bias. Publications regarding PI found to have higher asymmetry than BOP and CAL but less asymmetry than PPD.

**Figure 5:**
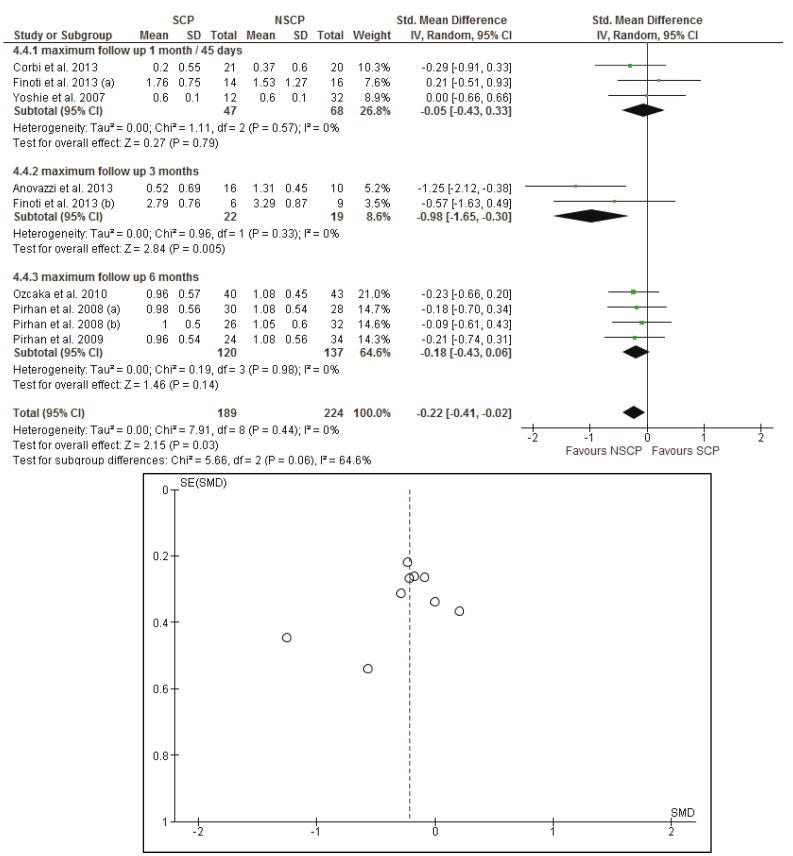
The forest plot and the funnel plot of the comparison: long term effects of each study and PPD (mm). Meta-analysis and forest plot comparing susceptible (SCP) and non-susceptible (NSCP) to periodontal disease with the difference of probing pocket depth (PPD) measurements long term. Presenting the mean differences of probing pocket depth (PPD) in 1 month-45 days, 3 months, 6 months separately and as total after non-surgical periodontal therapy. PPD is measured in millimeters (mm). The funnel plot is designed to check for the existence of publication bias. Publications regarding PPD found to have more asymmetry than the other clinical parameters.

Forest plots of the meta-analyses and funnel plots for assessing the publication bias, considering the long term effects of each study, for BOP, CAL, PI and PPD are presented in figures 2-5. Funnel plots (treatment effect against standard error), based on a visual and thus subjective assessment, look slightly asymmetric towards the bottom of the graph, which might be an indication of presence of publication bias.

## Discussion

Current results do not support the hypothesis that individuals with a genetic susceptibility to periodontal disease would have a worse clinical outcome after non-surgical periodontal therapy, compared to the non-susceptible individuals.

The number of the studies included in this systematic review was considered insufficient and inappropriate for the extraction of inferences regarding the evaluation of the tooth loss after non-surgical therapy, gingival bleeding index (GBI) and gingival index (GI). Bleeding on probing (BOP), clinical attachment loss (CAL), probing pocket depth (PPD) and plaque index (PI) were assessed to extract the conclusions.

The sample was limited to periodontal patients with no systematic diseases, in order to minimize the factors that can influence the periodontal treatment outcome. The clinical outcome was evaluated at least four weeks after completion of therapy. Concerning the type of therapy, studies with non-surgical periodontal treatment were only included. Nonsurgical periodontal treatment is the therapy of choice for moderated and advanced chronic periodontitis. Surgical treatment or anti-infective therapy could have influenced the clinical outcome and their results could not be compared with those of non-surgical treatment.

Based on the presented evidence there is an association between the susceptibility to chronic periodontal disease and the PPD in the first three months after non-surgical periodontal treatment and in long-term results. The analyses of the available data for each of the outcome measurements (BOP, CAL and PI) and for several post-intervention time frames (30-45 days, 3 months and 6 months) concluded that there is no statistically significance between susceptible and non-susceptible individuals. The heterogeneity of the included studies was found to be minimal and low to moderate.

PPD measurements between the baseline and three months after periodontal treatment were found to be statistically significant (*p*=0.009). The significance was resulted from only three studies with low to moderate heterogeneity (I2=28%) and thus the current evidence is insufficient to support the initial hypothesis. Long-term results of PPD measurements were statistically significant (*p*=0.03) with minimal heterogeneity (I2=0%).

An association was found between the presence of IL-1 susceptible genotype and smoking with the risk of tooth loss. However, only one study which investigated the effectiveness of the periodontal treatment in relation to tooth survival was included in this systematic review. McGuire and Nunn concluded that in case an IL-1 positive genotype of a patient is diagnosed, dentists should be suspicious for a greater likelihood of tooth loss. In this retrospective study, the prognosis of tooth survival in IL-1 positive nonsmokers was found to be less favorable than in IL-1 negative nonsmokers. However, providing periodontal therapy in combination with supportive periodontal treatment in IL-1 positive nonsmokers result in maintaining the teeth for many years (49).

The association between gene polymorphisms in chronic periodontitis and response to treatment was examined in patients that presented a susceptible genotype in the following genes IL-1, IL-4, IL-8, MMP-13, MMP-1 and MBL.

- Other systematic review

So far a systematic review was conducted in 2007 (50) which investigated the influence of the composite genotype in periodontitis progression and periodontal treatment outcome. This study compared only IL-1 different genotypes (presence of the allele 2 in the gene clusters IL-1A -889 and in IL-1B +3953). The heterogeneity of the study design, the periodontal status and the treatment plan of the included studies did not allow a meta-analysis to be done. The eleven included publications of the systematic review of 2007 led to the conclusion that there is inadequate evidence to determine an association among IL-1 positive genotype progression and treatment of periodontal disease outcomes.

- Site-based, tooth-based or subject based analysis

All the included studies used site-based and subject-based analyses of the clinical measurements. Although clinically it is more beneficial to conduct site-based and tooth-based analyses than subject-based, it is not correct to treat sites and teeth individually. Each site or teeth share the same conditions and risk factors.

A result of the differences between the site-based and the patient-based analyses is depicted in the levels of the periodontopathogens in the study of Finoti *et a*l. (43). In the site-based analysis a significant association between the susceptible group of patients compared to the other non-susceptible group was concluded. On the other hand, the multilevel logistic regression analysis, which is a patient-based analysis, did not report any statistically significant difference.

Functionality of gene polymorphisms at a protein level

Gene mutations can have significant, minimal or no phenotypic effect. Functional mutations can lead to changes in the structure of an encoded protein, or to a decrease, to or complete loss in its expression. For example, gene mutation can result in a substitution of a different aminoacid into the polypeptide sequence, or in the formation of a stop codon, which is going to give a truncated protein with little or no function. On the other hand, silent mutations result in the same aminoacid sequence of the original protein and as a result have no effect on the function of the protein.

From the papers included in the study, it is know that the mutations in the IL-8 gene are functional (43,46). Specifically, there is evidence that carriers of the single nucleotide substitution -251 (T/A) express higher levels of IL-8 in whole blood stimulated with E. Coli lipopolysaccharide (LPS) than individuals with the wild type allele (51). Moreover, an indication that the AGT/TTC mutation is functional, is the fact that patients carrying this mutated IL-8 allele harbor higher levels of red complex bacteria compared to diseased sites of ATC/TTC (43).

Moreover, for the mutations in exon 1 of MBL gene in the paper by Ozcaka *et al*. (22), there is evidence that they are functional, since they result in functional deficiency of the protein due to impaired oligomerization (52,53).

MMPs are enzymes that degrade proteins of the connective tissue and basements membranes and in periodontal disease play an important role in tissue destruction. It is interesting that the activity of MMPs is not regulated only in transcriptional level. All MMPs exist in a latent and active form. Disruption of a Cys-Zn2 bond, caused by chemical or physical means, results in a conformational change of the protein (54). This new ‘open’ conformation of the protein is active (54). MMP activity is regulated at a different level too. Tissue inhibitors of metalloproteinases (TIMPs) are enzymes that form noncovalent bimolecular complexes with the active forms of MMPs. TIMPS inhibit the activity of the fully competent MMP, but they can also delay or inhibit activation of the MMP proenzyme (55,56). In the studies by Pirhan *et al*. (44,45), the mutant allele formed by a G insertion at the polymorphic site of MMP-1 promoter binds more recombinant Ets-1 transcription factor and has significantly higher transcriptional activities compared to the wild type allele, which indicates that this insertion mutation is fully functional (57). As mentioned above, the increased transcriptional activity does not necessarily mean an increased destructive activity in connective tissue and basement membranes (44,45).

As far as the IL-4 studied polymorphism by Anovazzi *et al*. (41) and Finoti *et al*. (42) it was shown in a previous study that Th cells homozygous for the polymorphic allele produce lower levels of IL-4 (58). It is interesting also that the three mutations in the IL-4 gene are in linkage disequilibrium with each other and this is the reason why they are usually inherited all three of them together.

Yoshie *et al*. (48) studied the salivary enzyme levels after scaling and root planing in association with the IL-1 genotypes. The IL-1 polymorphism included in this research are functional, since in a previous study it was shown that carriers of the mutated allele were associated with a fourfold increase in IL-1a protein in gingival crevicular fluid (GCF) compared to healthy individuals (59).

The only study that does not provide evidence that the studied polymorphism is functional is the paper by D’Auito *et al*. (47) regarding the IL-6 polymorphism and its effect on periodontal treatment results. Overall, there is strong evidence that all the polymorphisms included in this meta-analysis have a significant effect at protein level.

- Limitations and future studies

One of the main limitations of the included studies was the number of patients. The study populations were characterized as small with different smoking habits. In the study of Finoti *et al*. (42), fifteen patients were enrolled and only six of them were susceptible to periodontal disease. Anovazzi *et al*. (41) also in another study included 26 subjects diagnosed with chronic periodontitis and 16 of them were IL-4 positive genotyped. The sample of the studies may thus affect in the reliability of the results. The results of the present meta-analysis give some indications of the influence of several gene polymorphism on the non-surgical periodontal treatment. The small sample size in some included studies as well as the limited studied gene polymorphisms help in understanding the tendency of such studies.

Furthermore, the calculated estimations of the CIs of the difference for each time interval are totally based on the assumption that the correlation between the two measures is 0.5, given that the studies did not report such information. Thusly, current results should be used with consideration.

Future studies should investigate the influence of different genetic polymorphisms of a variety of genes on several periodontal diseases and their association with the response of the non-surgical periodontal treatment. Genetics play a complex role in multifactorial diseases such as periodontal disease (60). It is possible that ethnicities and races are affected by different bacterial strains. The bacterial biofilm composition in combination with a certain susceptible genotype might affect the periodontal treatment outcome in certain populations. For example, it is known that A. actinomycetemcomitans serotypes vary among different ethnicities and geographic populations (61). This bacterial strain in a susceptible genotype background might change the periodontal treatment outcome.

There is a lot of evidence regarding the genetic susceptibility to periodontal diseases. The rejection of the hypothesis of this systematic review might be due to the multifactorial etiology of periodontal diseases. Predisposing factors such as cardiovascular diseases, rheumatoid arthritis and diabetes mellitus are possible causes of the absence of statistically significant differences between the susceptible and non-susceptible subjects to periodontal disease.

Genomic testing has fueled expectations for more effective prevention, earlier diagnosis, better prognosis and guide for the best individualized treatment protocol. Genetic testing has also promised a more successful treatment outcome for patients (62). The expectations became higher when the personalized dentistry presented to be the future of dentistry (63). Susceptible genotypes of periodontitis may have an important role in the onset and progression of periodontal disease, but their influence in non-surgical periodontal treatment outcome is non-significant.

The results of this meta-analysis indicate that more methodologically sound studies are necessary in the future to be conducted in order to obtain more reliable results. More studies in different ethnicities and races and in patients with systematic diseases that are associated with periodontal diseases are needed.

-Design of the studies-Quality assessment and risk of bias All the included studies are prospective cohorts. In such studies the exposure has already occurred, and the aim of these studies is to examine the outcome. The risk factors are determined in the beginning of the study, whereas the outcome will be recorded at a future time during the follow up. In prospective cohort studies, the selection bias of the population is not expected to be present. Unfortunately, the retention of subjects may bias the results. Cohort members may refuse to continue to participate in the study, which may lead to overestimation or underestimation of the study results. The included studies were assessed by the NOS ( Table 1), revealing that all the included studies were of high methodological quality and the risk of bias was low ( Table 4). The Quality in Prognostic studies (QUIPS) tool was also utilized to identify biases in the included studies (64). For the evaluation of the validity and bias of the studies, six domains were considered: study participation, study attrition, prognostic factor measurement, confounding measurement, outcome measurement, statistical analysis and reporting (64). The vast majority of the included publications exhibited low risk of bias for all the above criteria ( Table 5), validating the results of the Newcastle-Ottawa scale regarding the high methodological quality of the studies.

**Table 5:**
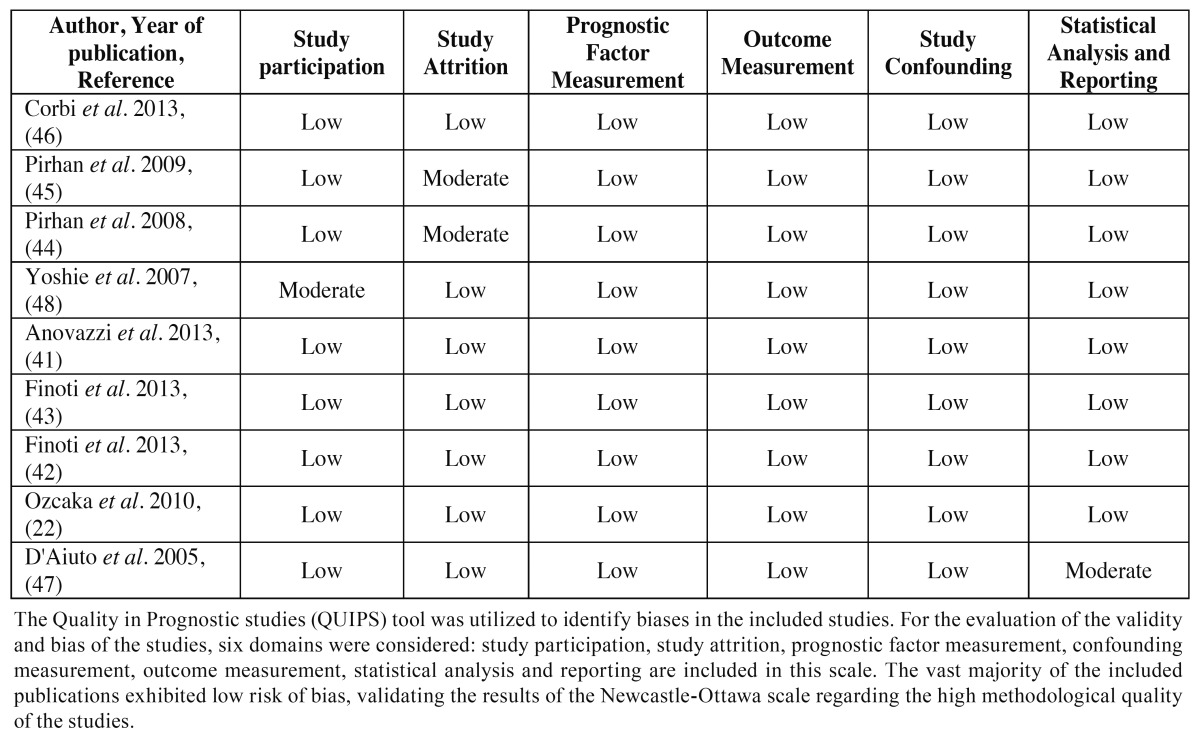
Quality in Prognostic Studies (QUIPS) tool.

## Conclusion

Within the limitations of this review, we conclude that the clinical outcome of the three examined parameters (BOP, CAL and PI) following non-surgical periodontal therapy has no difference regarding the genotype status of the patient. PPD measurements in the first three months and in long-term results found to have a significant difference with the baseline records. The existing literature does not allow us to reach a clear conclusion as far as the influence of smoking habits on the clinical outcomes after non-surgical periodontal therapy in susceptible individuals to periodontitis. More publications with focused questions are needed to identify a cause-effect relationship.
